# Supplementary material for: MetaRibo-Seq measures translation in microbiomes
Source: Nat Commun. 2020 Jun 29;11:3268. doi: 10.1038/s41467-020-17081-z (PMC7324362; doi:10.1038/s41467-020-17081-z)
Supplement: Supplementary file 10 — Supplementary Data 7 [file 41467_2020_17081_MOESM10_ESM.zip › File2/Confidence_High_Taxonomy/394858_out.krona.html]

Javascript must be enabled to view this page.

members
magnitude
magnitudeUnassigned
count
unassigned
taxon
rank

394858\_out

80

2
superkingdom
80

1224
phylum
1

28211
class
1

1
1948438
species

SRS013158\_contig\_number\_contig-100\_1.40018

79
1239
phylum

186801
class
79

79
order
186802

1
family
541000

1
genus
1263

species
46228

SRS1041090\_contig\_number\_contig-100\_2730.2731
1

78
186803
family

841
genus
78

species
166486

SRS011084\_contig\_number\_3254SRS013940\_contig\_number\_15718SRS013951\_contig\_number\_17217SRS014287\_contig\_number\_19996SRS014613\_contig\_number\_13485SRS014855\_contig\_number\_480SRS014923\_contig\_number\_25554SRS014948\_contig\_number\_18655SRS015065\_contig\_number\_28723SRS015190\_contig\_number\_10022SRS015264\_contig\_number\_7208SRS015431\_contig\_number\_32111SRS015578\_contig\_number\_70SRS015579\_contig\_number\_10401SRS015854\_contig\_number\_contig-100\_630.127074SRS015890\_contig\_number\_23175SRS015960\_contig\_number\_4556SRS017821\_contig\_number\_10185SRS018623\_contig\_number\_11512SRS019068\_contig\_number\_34114SRS019267\_contig\_number\_636SRS019397\_contig\_number\_883SRS024435\_contig\_number\_26018SRS024549\_contig\_number\_5554SRS043001\_contig\_number\_9402SRS043411\_contig\_number\_contig-100\_813.78042SRS043701\_contig\_number\_15734SRS045739\_contig\_number\_9537SRS049900\_contig\_number\_13220SRS050422\_contig\_number\_14173SRS050925\_contig\_number\_33003SRS050941\_contig\_number\_17919SRS052027\_contig\_number\_4294SRS053335\_contig\_number\_9267SRS054956\_contig\_number\_contig-100\_1041.90428SRS055017\_contig\_number\_16021SRS056273\_contig\_number\_32941SRS056519\_contig\_number\_18346SRS058070\_contig\_number\_331SRS063040\_contig\_number\_16573SRS063518\_contig\_number\_3626SRS065504\_contig\_number\_23892SRS075341\_contig\_number\_14837SRS075716\_contig\_number\_4414SRS076756\_contig\_number\_3072SRS077454\_contig\_number\_12770SRS077641\_contig\_number\_contig-100\_5396.101912SRS098061\_contig\_number\_13112SRS098644\_contig\_number\_1299SRS098827\_contig\_number\_10879SRS098881\_contig\_number\_24515SRS104084\_contig\_number\_10579SRS1041130\_contig\_number\_4821SRS104485\_contig\_number\_655SRS105153\_contig\_number\_33449SRS1055034\_contig\_number\_10563SRS1055067\_contig\_number\_contig-100\_715.41657SRS1055069\_contig\_number\_contig-100\_656.37847SRS142503\_contig\_number\_28380SRS142781\_contig\_number\_9176SRS143070\_contig\_number\_15909SRS143148\_contig\_number\_contig-100\_383.84811SRS143417\_contig\_number\_13492SRS143598\_contig\_number\_21697SRS143722\_contig\_number\_15571SRS143780\_contig\_number\_contig-100\_1323.191996SRS144183\_contig\_number\_15783SRS144362\_contig\_number\_38791SRS144714\_contig\_number\_25133SRS147919\_contig\_number\_20599SRS148091\_contig\_number\_3581SRS148159\_contig\_number\_40033SRS148784\_contig\_number\_25710SRS149181\_contig\_number\_24278SRS149879\_contig\_number\_25169SRS893252\_contig\_number\_446SRS893378\_contig\_number\_16819
77

1

SRS018984\_contig\_number\_26778
species
360807
